# Hospital Festivals and Meetings

**Published:** 1894-12-29

**Authors:** 


### HOSPITAL FESTIVALS AND MEETINGS.

### METROPOLITAN HOSPITAL SUNDAY FUND.

This fund held its annual general meeting on Monday, December 18th, at the Mansion House, the Lord Mayor presiding. The meeting was well attended, and amongst others present were Lord Knutsford, Mr. C. T. Ritchie, Mr. R. Biddulph Martin, M.P., Canon Ingram, the Chief Rabbi, the Rev. C. J. Ridgeway, Canon Murnane, and Mr. H. N. Custance (secretary).

After a few words from the Lord Mayor the adoption of the report of the council for 1894 was moved by Mr. Ritchie.

Last year a falling off in the subscriptions from £41,500 to £39,200 had to be deplored, but this year the council had to congratulate themselves upon an increase, the amount collected reaching the sum of £43,600, the largest total, save one, since the fund was started. The number of congregations subscribing had also increased from 1,772 to 1,799. Mr. Ritchie thought all would agree with Canon Fleming, who in writing to regret his inability to attend the meeting had expressed a hope that every effort would be made to augment the number of subscribing churches and chapels. The fund needed growing supplies, because though its increase was most satisfactory the demands upon it grew ever greater, and the revenue collected was not sufficient to cover the expenditure of the hospitals. He thought it would be well if the fund could be placed upon such a footing that it might not be necessary to use the whole of the legacies received during the year, but that some might be put by to form a permanent revenue-bearing fund for the benefit of the hospitals. It was unsatisfactory to find that so many beds in the principal London hospitals were vacant for want of funds.

An increased number of surgical appliances had been given away in 1894, conferring immense benefit upon many. Mr. Ritchie laid special stress on the value of the work done by the hospitals in the training of nurses, of whom he understood that no fewer than 1,200 were available for attendance upon the public at large. He thought that credit was due to the fund for the care taken to investigate the accounts and expenditure of the various institutions. The cost of management of the fund itself had this year been only 3·6 per cent., a lower average than for the last twenty-two years. Mr. Ritchie concluded by cordially thanking all those who had helped the fund to carry on its good work.

The Rev. Dr. Kennedy, who seconded the motion, said he could not agree with Mr. Ritchie's suggestion as to an accumulated fund. He thought such a plan would militate against large collections.

Resolutions were then passed re-electing for 1895 the council of 1894, with the Rev. C. H. Grundy, vicar of St. Peter's, Brockley, to fill the vacancy caused by the death of Canon Rowsell; Canon McCormick in the place of Canon Pelham, who has left London; and Sir George R. Tyler in the place of Sir S. Crossley, who has been elected to a seat on the committee of distribution; and fixing June 16th as Hospital Sunday for 1895.

### INSTITUTIONS FOR THE BLIND.

An Address by Mr. Burdett.

The annual distribution of prizes to the successful pupils of the London Society for Teaching the Blind to Read took place on Saturday at the society's head-quarters, 10, Upper Avenue Road, Swiss Cottage. Mr. Henry C. B[illegible]dett presided, and among those present were Mr. [illegible]. K. Blyth (deputy chairman of the committee), Mr. Loveland-Loveland (honorary secretary), Major General Young, Dr. Walford, and Captain Webber, R.N., secretary. In opening the proceedings, Mr. Blyth said that highly satisfactory work had been accomplished during the past twelve months. The society had adopted the recommendations of the Royal Commission on the Teaching of the Blind, which were to the effect that the blind should receive such instruction as would enable them to gain a livelihood on leaving any particular institution. The number of workshops had been increased during the past year, and one for the girls' department had been erected for the purpose of enabling them to practice and study the art of chair and basket making. A tuning-room was also an addition to the institution, and formed a branch in which work was taught which would be very useful to the blind in after life. (Applause.) They were beginning to hope that the technical education undertaken by the London County Council, and for which they were giving grants to other schools, would be extended to that institution. They had brought the matter before the notice of the County Council, with view to receiving, if possible, support from them. (Applause.)

Mr. Loveland-Loveland then read the inspectors' reports with reference to general education, music, and Scripture. Mr. F. J. Wrothesley, in his report on the general education, expressed himself eminently satisfied with the result of the examination so far as the girls were concerned With regard to the boys' department, he regretted that his report could not be so satisfactory. He advised a thorough revision, as, owing to music and technical work, there would not be so much time devoted to other subjects as was compatible with efficiency. The report of the Rev. J. W. Bennett on Scriptural work, and Mr. C. W. Pearce on the work of the music pupils were also of a satisfactory character.

Mrs. BURDETT having distributed the prizes to the successful students, Major-General YOUNG moved, and Mr. SPILLER seconded, a vote of thanks to the examiners. Thanks were also accorded to Mr. and Mrs. Burdett on the proposition of Mr. BLYTH, seconded by Mr. F. O. SMITHERS.

Mr. BURDETT said it had been his lot some time since frequently to pass the institution and to wonder as to the work carried on therein, but he had never seen the inside until the previous day, and this led him to the thought how much for their advantage it would be if the dwellers in this great wilderness of London daily passing such institutions and living next door to each other, could become better acquainted with one another's lives. He felt that the visit of inspection of the day before, made that he might not occupy the chair without some special knowledge of the work of the school, had been a most gratifying and helpful one, a visit he should not forget, and one he should profit by for the rest of his life. Would that everyone might visit these institutions which provide for those less fortunate than themselves, and so learn a lesson in sympathy, in seeing what has been done and what it is possible to do to help others in the wisest way.

SYSTEMS OF VOTING AND MANAGEMENT.

The work of the institution was many-sided; examination of its financial aspect showed that to be in all respects sound, under the management of Captain Webber, a distinguished officer of Her Majesty's Navy, whose knowledge and experience enabled him to make the position of the institution one of lasting strength. The general condition of the children, their bright and happy faces, must give pleasure and satisfaction to those who understood such signs. Mr. Burdett, in criticising the method of admission by vote, spoke strongly against the system as disgraceful in itself, and not charity at all, and said he was glad to find that the society had gone half way towards putting a stop to it in their case by insisting upon the voting papers being returned to the committee for each case to be dealt with on its merits. The Clergy Orphan Society adopted a regulation by which voting papers were invalid unless returned to the secretary's office a week before the election. If anyone wanted to know what traffic in votes meant, let them attend an election at the Cannon Street Hotel, where no greater travesty of horrors could be seen. Blind institutions, on the whole, could be taken as amongst the best managed charities in this country. The larger institutions, with an expenditure of £15,000 are managed at a cost of 5 per cent.; the smaller ones, with an expenditure of from £3,500 to £7,000, at a cost of 10 per cent., the cost of management generally being 100 per cent. cheaper than at other institutions. Alluding to pension societies, Mr. Burdett said he should like to see one great consolidated fund for this purpose, whereby the management expenses would be much less than under the present multiple system.

BOOKS FOR THE BLIND.

As a maker of books himself, Mr. Burdett said he had been deeply interested in visiting the room where the books were kept and in realising what books for the blind actually meant. For instance, an ordinary small Christmas annual, if printed for their use, would make two huge imperial quarto volumes. The whole Bible meant fifty-two such volumes, while the Psalms would fill seven. And what could be said to enhance the glory of the man (M. Haüy) who first invented a system by which the blind could read, not only printing, but music? Now a monthly magazine was published for the use of the blind, numbering some 30,000 in this country. It was originated by a Mr. Taylor, F.R.S.

EDUCATION OF THE BLIND.

With regard to the education of the blind, it was strange and sad that hitherto the nation had taken little concern in the matter, and efforts in that direction were left to the enterprise of private individuals, and even now the State was taking a hand in the work, Mr. Burdett thought the prospect was not a very bright one, because it was to be feared that the supreme importance of technical education might not be adequately realised by those charged with the duties of superintendence and examination. He should like to urge Mr. Acland to inspect these schools himself, in order to see that proper time-tables might be prepared, and the school-day divided proportionately between technical and general education. It was to be hoped that Mr. Acland might be induced to take the matter up and see that proper guidance was given in this direction, thus avoiding friction where it would be most undesirable.

WORK AND THE POST OFFICE CONTRACTS.

Touching upon the kinds of work suitable for the blind, Mr. Burdett said he believed that piano tuning was the most profitable occupation of all, blind people often having an excellent ear for music. They were great, too, at basket-work and chair-making, and he had been himself much struck at witnessing the repair of a large elaborately-made deck chair by two blind workers, the chair in question, moreover, not having been made by them in the first instance. It would be turned out of the workshop as good as new, which seemed to him very marvellous. The girls excelled at knitting and crochet. With regard to the sale of the work, it ought to be placed on record that the secretary stated owing to a reported system of blackmailing on the part of some underling at the Post Office, the society could have no dealings with it, as it had with some of the great railway companies in this country. If this were really so it was a disgrace to the nation. Mr. Burdett said he understood that the facts had been brought to the knowledge of the Post Office by the authorities of one of these schools recently. Such a question as this was one upon which Mr. Burdett, for his part, would be prepared to turn out a Government, and it was not too much to say that Mr. Morley must either put a stop to such an abuse, or be prepared to go. A system such as this was what had brought China to its present condition. For the higher grades of work, the blind excelled in music and singing, and it was worthy of note that there was an entire band of blind people at Norwich, which had made a cosiderable sum of money only last year. Typewriting now was offering another opening, and the blind were also becoming able to take up medicine in a small way in the direction of massage. At the Edinburgh Infirmary it was now under consideration to teach the blind the art of massage, and allow them to practise in the infirmary and employ them when taught, The decision of the Edinburgh authorities would be awaited with interest.

BLIND HEROES AND THEIR LESSON.

Mr. Burdett went on to draw a pathetic picture of the contrasts between those who enjoy the untold blessings of sight and those who are deprived of the light of the sun, enumerating examples of well-known men who had risen superior to their afflictions. Milton, who wrote the first history of England before the Conquest, ever published, compiled from the Saxon Records after he was blind; Handel, the musician; Henry Fawcett, the politician, who actually enjoyed the pleasure of fly-fishing after he was deprived of sight; Dr. Robertson, of Brighton, member of Parliament for many years after blindness came upon him; and James Holman, the blind traveller, who wrote an account of his journeyings and the impression received. All these examples showed how, with courage and hope, even to blind people all things were possible; and it was a fact to record that the blind were usually bright and happy, while deaf people were more or less irritable. Mr. Burdett concluded his speech, which was listened to with much appreciation, by hoping that those present would have a bright memory of the day not as a mere function, and that the occasion might stir up them to work in the world with hearts as well as hands, and to go forward with courage and hope. On behalf of Mrs. Burdett and himself he thanked them all for their welcome.

A short musical programme was afterwards carried most successfully through, the pupils doing much credit to their teachers, and evidently, too, thoroughly enjoying the music. The part songs were especially good.

### THE WESTMINSTER HOSPITAL—MEMORIAL TO DR. STURGES.

A numerously attended meeting was held on Wednesday, December 19th, in the board-room of Westminster Hospital, to raise some memorial to the late Dr. Octavius Sturges, the Senior Physician. In the absence of the Duke of Westminster (the President), who wrote to express his regret at not being able to attend in consequence of his absence from London, the chair was occupied by Mr. George Cowell, F.R.C.S., Senior Surgeon to the hospital. Among those present were Sir Rutherford Alcock, the Dean of Westminster, the Rev. Dr. Troutbeck, Mr. H. D. Erskine (of Cardross), Dr. Donkin, Dr. J. B Potter, Dr. de Havilland Hall, Mr. W. G. Spencer (Dean of the Medical School), and a large number of past and present students of the hospital. Letters expressing sympathy with the object of the meeting having been read from Dr. W. B. Cheadle, Dr. Whipham, Sir Benjamin Ward Richardson, Dr. W. Howship Dickenson, Dr. Thorowgood, Dr. Sidney Coupland, Dr. F. S. Palmer, Mr. F. S. Lambert, and many others, the following resolution was proposed by Sir Rutherford Alcock seconded by the Rev. Dr. Troutbeck, and carried unanimously, viz.: "That this meeting cordially approve of the proposal to establish a memorial to Dr. Sturges, and pledges those present to make every exertion to promote this object." The second resolution, proposed by the Dean of Westminster, seconded by Dr. J. B. Potter, and supported by Mr. W. G. Spencer and carried unanimously, was as follows, viz.: "That the memorial be a prize, exhibition, or scholarship in connection with the Westminster Hospital Medical School, with which Dr. Sturges was so long associated, a portion of the money raised being set aside for the purchase of four permanent photographic portraits of Dr. Sturges, and to cover the cost of a small photograph to be issued with the forthcoming volume of the hospital reports." On the motion of Dr. A. M. Gossage, seconded by Mr. Thomas Bond, a third resolution was then carried for the formation of a large and representative committee for the purpose of carrying out the former resolution, after which the meeting dispersed.

